# Antibacterial Activity and Mechanism of Action of Whey Protein-ε-Polylysine Complexes against *Staphylococcus aureus* and *Bacillus subtilis*

**DOI:** 10.3390/foods11152311

**Published:** 2022-08-02

**Authors:** Yuecheng Meng, Li Lou, Zhipeng Shao, Jie Chen, Yanhua Li, Tianqi Zhang

**Affiliations:** 1 School of Food Science and Biotechnology, Zhejiang Gongshang University, Hangzhou 310018, China; myczjgsu@gmail.com (Y.M.); 1902008085@pop.zjgsu.edu.cn (L.L.); chenjie@zjgsu.edu.cn (J.C.); 21020080035@pop.zjgsu.edu.cn (T.Z.); 2 Research and Development Center, Wuxi Biortus Biosciences Co., Ltd., Jiangyin 214437, China; 15010000019@pop.zjgsu.edu.cn

**Keywords:** whey protein-ε-polylysine complexes, Gram-positive bacteria, antibacterial activity, antibacterial mechanism

## Abstract

ε-Polylysine (ε-PL) is a cationic antimicrobial peptide, which easily forms complexes with food polyanions to weaken its antibacterial activity. A whey protein-ε-PL complex delivery system was found to be able to solve this problem. This study investigated the antimicrobial activity of the complexes and their mechanism against Gram-positive bacteria. The minimal inhibitory concentration of the complexes with different ε-PL contents against *Staphylococcus aureus* and *Bacillus subtilis* were 19.53–31.26 and 3.90–7.81 μg/mL, respectively, which were similar to free ε-PL. Furthermore, the whey protein-ε-PL complexes had a strong bactericidal effect on *Bacillus subtilis*. The inhibition zone diameters of the complexes against *Staphylococcus aureus* and *Bacillus subtilis* containing 5000 μg/mL of ε-PL were 14.14 and 16.69 mm, respectively. The results of scanning electron microscopy showed that the complexes could destroy the cell membrane structure in *Bacillus* *subtilis*, resulting in holes on the surface, but not in *Staphylococcus aureus*. The results of molecular dynamics simulation showed that under electrostatic interaction, the complexes captured the phospholipid molecules of the bacterial membrane through the hydrogen bonds. Parts of the ε-PL molecules of the complexes were embedded in the bilayer membrane, and parts of the ε-PL molecules could penetrate the bilayer membrane and enter the bacterial internal environment, forming holes on the surface of the bacteria. The antibacterial results in fresh meat showed that the whey protein-ε-PL complexes could reduce the total mesophilic and *Staphylococcus aureus* counts. This study on the antibacterial activity mechanism of whey protein-ε-PL complexes could provide a reference for the application of ε-PL in protein food matrices.

## 1. Introduction

ε-Polylysine (ε-PL) is a potent natural antibacterial agent widely used for the preservation of dishes, noodles, cheese, and other foods [1,2,3,4,5,6]. ε-PL is an extracellular substance of *Streptomyces albulus* ssp. *lysinopolymerus* [5,7,8], consisting of 25 to 30 L-lysine residues linked by amide bonds formed by ε-amino and α-carboxyl groups [9]. More importantly, it has been confirmed that ε-PL has the good properties of water solubility, thermal stability, non-toxicity, and antibacterial activity [9,10,11].

It is worth mentioning that ε-PL is a kind of positively charged polymer, and, therefore, it can be adsorbed by the negatively charged cell surface through its cationic properties, leading to the formation of cellular holes and the disruption of morphology [12,13,14]. However, ε-PL has encountered some bottlenecks in practical application. Positively charged amino groups also form insoluble precipitates with anions in the food matrix, thereby reducing the antimicrobial activity of ε-PL [15,16,17]. It was found that the optimum addition level of ε-PL in protein-rich foods was far greater than that in starch products [18]. Additionally, excessive addition could bring astringency to the products and increase the product cost. To solve these problems, the combination of cationic ε-PL and anion to form nanoparticles was found to achieve good solubility while retaining its antibacterial activity [16,19,20]. Many studies have focused on the use of ε-PL in combination with other components, especially polysaccharides. For example, the ε-PL-pectin complexes were reported to achieve a good balance between good solubility and antibacterial activity [19,21]. Other researchers also found that ε-PL-chitosan-sodium alginate nanoparticles had three times the in vitro antibacterial activity of free ε-PL [16]. ε-PL could covalently bind to dextran and the complex not only retained the bacteriostatic properties of ε-PL but also exhibited good emulsifying properties [22].

Similar to polysaccharides, complexing ε-PL with proteins to form nanoparticles is also an effective strategy. In particular, whey protein is a kind of negatively charged polymer that can be combined with cationic polymers of ε-PL by interaction. It was found that the complexes formed by ε-PL and whey protein still had antibacterial activity against *Salmonella enteritidis* [23]. In addition, it was elucidated that the whey protein-ε-PL complexes could form holes on the bacteria surface, resulting in the inactivation of *Escherichia coli* in sauce duck [24]. Therefore, it is of great significance to study the antibacterial activity mechanism of whey protein-ε-PL complexes in other microbial groups in food products. The purpose of this study was to investigate the antibacterial activity of whey protein-e-PL complexes against Gram-positive bacteria (*Staphylococcus aureus* and *Bacillus subtilis*) and test their application in protein food matrices.

## 2. Materials and Methods

### 2.1. Sample Preparation

Food-grade whey protein and ε-PL were purchased from Hilmar Cheese Company and Zhejiang Yinxiang Company, respectively. Table 1 shows the components and properties of whey protein. *Staphylococcus aureus* (ATCC 25923) and *Bacillus subtilis* (ATCC 11778) were purchased from Huankai Microbial Co., Ltd., which were cultured in Mannitol Salt Broth/Agar or Nutrient Broth/Agar, respectively (Hangzhou Microbial Reagent Co., Ltd., Hangzhou, China). The final concentration of the two tested strains in the broth cultures after reaching the stationary phase was 1 × 10^8^ CFU/mL.

The samples were prepared according to previous studies [24,25]. Briefly, the sample powders were dissolved in distilled water to prepare whey protein solution (10 mg/mL) and ε-PL solution (10 mg/mL), respectively. The whey protein solution, ε-PL solution, and distilled water were mixed in different proportions to prepare complex solutions with different ratios of whey protein to ε-polylysine mass (R_WP-PL_). The R_WP-PL_ were 50:0, 50:1, 50:2, 50:10, 50:20, 50:30, 50:40, and 50:50 (the final concentrations of ε-PL were 0, 100, 200, 1000, 2000, 3000, 4000, and 5000 μg/mL, respectively).

### 2.2. Determination of Minimum Inhibitory Concentration

The minimum inhibitory concentration (MIC) of whey protein-ε-PL complexes was determined using the broth microdilution method with minor modifications [26]. Briefly, the whey protein-ε-PL complexes with different R_WP-PL_ and ε-PL solution were diluted in 96-well plates with Mueller-Hinton medium in a 2-fold gradient. Then, 100 μL of the tested bacteria (*Staphylococcus aureus* or *Bacillus subtilis)* grown to logarithmic phase (1 × 10^5^ CFU/mL) was mixed with 100 μL of the samples. To the last two columns, *Staphylococcus aureus* or *Bacillus subtilis* without antimicrobial agent and Mueller-Hinton medium as controls were added to ensure bacterial viability and to detect contamination of Mueller-Hinton medium. The 96-well plates were incubated in a 37 °C incubator for 24 h. Growth was assessed by measuring OD at 600 nm and the MIC was determined as the minimum concentration at which wells did not show any OD increase.

### 2.3. Determination of Minimum Bactericidal Concentration

According to the method of Geng et al., the minimum bactericidal concentration (MBC) of whey protein-ε-PL complexes was determined [27]. Specifically, after the experiment of MIC, the bacterial suspension in the clear wells (100 μL) was inoculated on the solid medium. Then, the sample was cultured at 37 °C for 24 h.

### 2.4. Analysis of Inhibition Zone

The inhibition zone was determined by the Oxford cup method with slight modifications [28]. The Oxford cups were placed in the Petri dish. Then, the bacterial suspensions of *Staphylococcus aureus* and *Bacillus subtilis* were added into the sterilized plate counting agar medium, cooled to 45 °C and mixed well (the final bacterial concentration was 1 × 10^6^ CFU/mL), and appropriate amounts of medium with bacterial suspensions were added to the Petri dishes. After a while, the Oxford cups were removed, and 200 μL of the complexes in different proportions, ε-PL solution (5000 μg/mL), and whey protein solution (5000 μg/mL) were added to the wells. The same volume of sterile water was used as a blank control group. After culturing in a 37 °C incubator for 24 h, the diameter of the inhibition zone was determined.

### 2.5. Scanning Electron Microscopy (SEM) Analysis

To study the effect of the whey protein-ε-PL complexes on the bacterial cell structure, the cell morphology was observed using scanning electron microscopy (SU8010, Hitachi, Japan). *Staphylococcus aureus* and *Bacillus subtilis* were cultured to the stationary phase (1 × 10^8^ CFU/mL). According to MIC and MBC data, the concentration of ε-PL was 5000 μg/mL and the whey protein-to-ε-PL complexes (1:1 mass ratio of whey protein-to-ε-PL with a final ε-PL concentration of 5000 μg/mL) was chosen. Then, the two tested strains that reached the stationary phase were mixed with ε-PL solution or whey protein-ε-PL complexes at a volume ratio of 9:1. After mixing, the test samples were cultured at 37 °C for 2 h and then treated according to the method described by Ukuku et al. [29]. Lastly, the samples were observed using SEM.

### 2.6. Molecular Dynamics Simulation

Molecular dynamics (MD) simulation of the interaction of whey protein and ε-PL with the bacterial membrane of *Staphylococcus aureus* and *Bacillus subtilis* was performed using GROMACS 5.1.4 software package [30]. The Martini force field was employed in this study [31].

**Figure 1:**
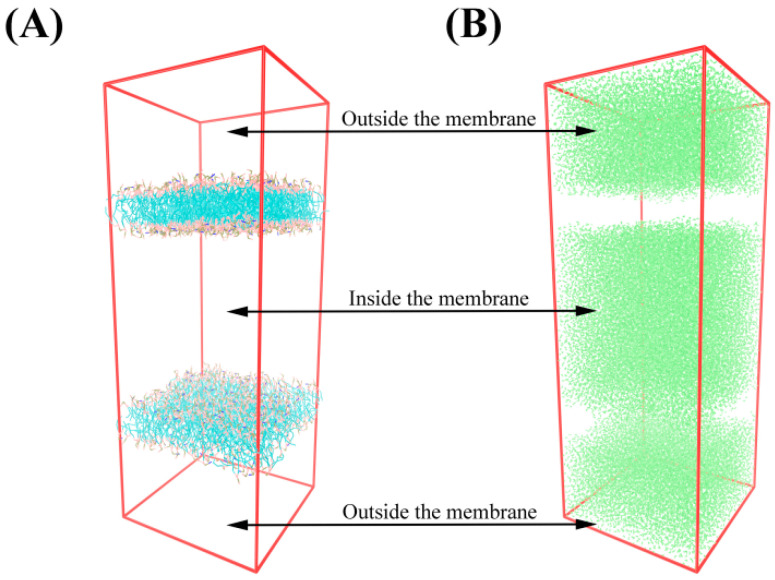
Bilayer model. (**A**) No water molecules. (**B**) Only water molecules.

Our previous study showed that the adsorption mechanism of the complexes on the membrane of Gram-positive bacteria (*Staphylococcus aureus* and *Bacillus subtilis*) was similar to that of Gram-negative bacteria (*Escherichia coli*) [24]. In order to elucidate the mechanism by which complexes destroy Gram-positive bacteria membrane, a double membrane model was established in this study (Figure 1). The Gram-positive bacteria bilayer membrane model was composed of POPE (1-Palmitoyl-2-Oleoyl-sn-Glycero-3-Phosphoethanolamine) and POPG (1-Palmitoyl-2-Oleoyl-sn-Glycero-3-Phosphoglycerol) molecules, in which the POPE and POPG molecule ratio was 1:3. The upper and lower phospholipid bilayer constituted the inner environment of bacteria while the outer membrane belonged to the outer environment of bacteria.

The molecular information on α-lactalbumin (PDB: 1HFZ) [32] and β-lactoglobulin (PDB: 3NPO) [33] and the major components of whey protein were provided by the RCSB Protein Data Bank. All topologies of ε-PL (degree of aggregation = 25), POPE, and POPG for GROMACS were generated by CHARMM-GUI [34] and then modified according to the Martini force field requirements.

In this study, six simulated systems were implemented (Table 2). The MD simulation box size was 12.5 nm × 12.5 nm × 48 nm, and the distance of both the upper and lower membranes from the top and bottom of the box was 10 nm. During model preparation, ε-PL, α-La (α-lactalbumin), β-Lg (β-lactoglobulin), ε-PL-α-La complex (ε-PL-α-lactalbumin), and ε-PL-β-Lg complex (ε-PL-β-lactoglobulin) were placed outside the bilayer membrane (outer environment). After adding polarizable water and counterions, 10 ns of MD simulation was performed to equilibrate the system while ε-PL, α-La, β-Lg, complex, and membrane were constrained. The simulated pressure and temperature were controlled at 1 bar and 37 °C, respectively [35,36]. The long-range electrostatic force was calculated using the particle grid Ewald method [37]. The cutoff for both Van der Waals and short-range electrostatic interaction was 11 Å. Periodic boundary conditions were applied. Other detailed information was available from CHARMM-GUI. Ultimately, 100–300 ns of MD simulation of the system was carried out.

### 2.7. Antibacterial Effect of Whey Protein-ε-PL Complexes on Total Mesophilic Counts and Native Staphylococcus aureus in Fresh Meat

Fresh meat was purchased from Yonghui supermarket (Hangzhou, China) and kept at 4 °C. Firstly, fresh meat was cut into 1 cm chunks. Secondly, fresh meat was divided and soaked in whey protein-ε-PL complexes (R_WP-PL_ = 50:50, the final concentration of ε-PL was 5000 μg/mL), ε-PL solution (5000 μg/mL, positive control), and whey protein solution (5000 μg/mL, negative control). At regular intervals, the total mesophilic counts and native *Staphylococcus aureus* in room temperature soaked fresh meat were determined according to the standard microbiological testing method [38].

### 2.8. Data Analysis

The experiments were repeated three times on different working days. Data were analyzed with one-way ANOVA followed by Turkey’s test (*p* < 0.05) and LSD test, and afterwards by IBM SPSS Statistics 22. The data were expressed as means ± standard deviation.

## 3. Results and Discussion

### 3.1. Minimum Inhibitory Concentration of Whey Protein-ε-PL Complexes

MIC is defined as the minimum concentration required for a bacteriostatic agent to inhibit microbial growth, and it is often used to test the bacteriostatic activity of potential antimicrobials. Table 3 shows the MIC of the ε-PL solution and whey protein-ε-PL complexes against *Staphylococcus aureus* and *Bacillus subtilis*. All the complexes with different mass ratios had good inhibition against *Staphylococcus aureus* and *Bacillus subtilis*. The MIC of the ε-PL solution and whey protein-ε-PL complexes against *Staphylococcus aureus* were 19.53 and 19.53–31.26 μg/mL, respectively, and against *Bacillus subtilis* were 4.88 and 4.88–7.81 μg/mL, respectively. Thus, the inhibitory activity of the complexes against *Bacillus subtilis* was stronger than that against *Staphylococcus aureus*. These results were probably caused by differences in the cell membrane between the tested strains, such as charges, amphoteric molecular structure, and hydrophobicity. These results were similar to previous reports [11,39], which indicated that the MIC of ε-PL against *Escherichia coli* and *Staphylococcus aureus* was 12.5 μg/mL, respectively. However, some research results were slightly different from the results of this paper, which might be related to differences in the tested strains [16,40]. More specifically, in this study, we found that the complexes retained the bacteriostatic properties of ε-PL, and the binding of ε-PL and whey protein did not weaken the antibacterial activity of the complexes.

### 3.2. Minimum Bactericidal Concentration of Whey Protein-ε-PL Complexes

MBC of whey protein-ε-PL complexes against *Staphylococcus aureus* and *Bacillus subtilis* is shown in Table 4. According to the method [27], if there is no colony growth in the culture medium, it means that the compound has a bactericidal effect on microorganisms; otherwise, it means that the compound has only an inhibitory effect on microorganisms. The MBC of the ε-PL solution and whey protein-ε-PL complexes against *Staphylococcus aureus* were 39.06 and 39.06–100.00 μg/mL, respectively, and against *Bacillus subtilis* were 9.77 and 9.77–25.00 μg/mL, respectively. It was found that the MBC of whey protein-ε-PL complexes against *Staphylococcus aureus* and *Bacillus subtilis* was about two to four times higher than its corresponding MIC. The whey protein-ε-PL complexes had a strong bactericidal effect on *Bacillus subtilis*, which showed the best bactericidal activity among the treatments as the mass ratio of whey protein-to-ε-PL was 1:1.

### 3.3. Inhibition Zone of Whey Protein-ε-PL Complexes

**Figure 2:**
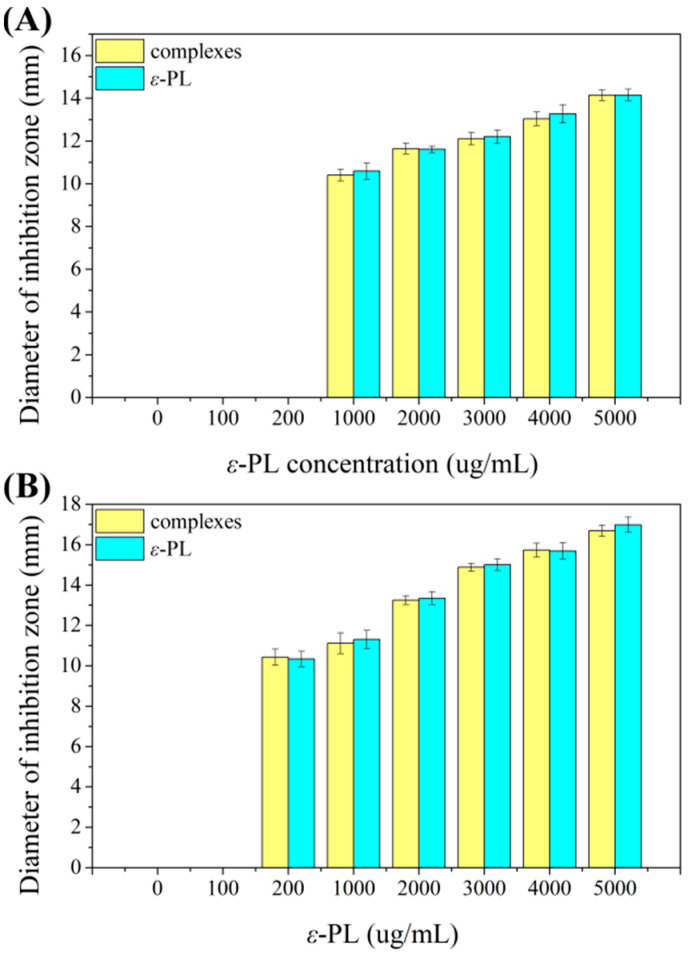
The inhibition zone diameters of ε-PL and whey protein-ε-PL complexes at different concentrations against (**A**) *Staphylococcus aureus* and (**B**) *Bacillus subtilis*.

The diameter of the inhibition zone can indicate the strength of the inhibition, where a larger inhibition zone means a more significant inhibition effect. The inhibition zone diameters of the ε-PL solution and the complexes against *Staphylococcus aureus* and *Bacillus subtilis* are shown in Figure 2. It was found that the inhibition zone diameters of the complexes against *Staphylococcus aureus* were in the range of 0–14.14 mm while the inhibition zone diameters of the ε-PL solution at the same concentration were in the range of 0–14.15 mm. The inhibition zone diameters of the complexes against *Bacillus subtilis* were in the range of 0–16.69 mm. Compared with the positive control, the inhibition zone diameters of the ε-PL solution at the same concentration were in the range of 0–16.99 mm. The results clearly indicated that the whey protein-ε-PL complexes retained the inhibitory effect of ε-PL on *Staphylococcus aureus* and *Bacillus subtilis*, and the binding of whey protein and ε-PL did not reduce the bactericidal effect of ε-PL. This is in line with the findings of Shao et al. [24]. This phenomenon could be explained as only part of the amino groups of ε-PL were bound to whey protein while the rest could still interact with the bacterial surface, resulting in bacterial death.

### 3.4. Morphological Changes of Staphylococcus aureus and Bacillus subtilis

**Figure 3:**
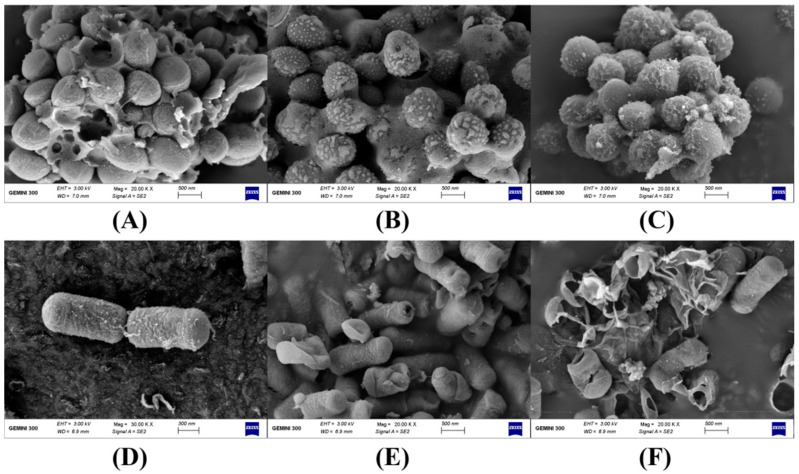
SEM images of *Staphylococcus aureus* and *Bacillus subtilis* exposed to different treatments. (**A**) Untreated *Staphylococcus aureus*. (**B**) *Staphylococcus aureus* treated by ε-PL. (**C**) *Staphylococcus aureus* treated by whey protein-ε-PL complexes. (**D**) Untreated *Bacillus subtilis*. (**E**) *Bacillus subtilis* treated by ε-PL. (**F**) *Bacillus subtilis* treated by whey protein-ε-PL complexes. The concentration of ε-PL was 5000 μg/mL and the mass ratio of whey protein-to-ε-PL was 1:1.

To study the effect of whey protein-ε-PL complexes on the bacterial cell membrane structure, the morphological changes in *Staphylococcus aureus* and *Bacillus subtilis* treated with the complexes were observed using SEM (Figure 3). As shown in Figure 3A, the surface of untreated *Staphylococcus aureus* was smooth, and the bacteria body was complete and round with distinct boundaries. After treatment for 2 h at 37 °C with ε-PL (5000 μg/mL) and the complexes (1:1 mass ratio of whey protein-to-ε-PL with a final ε-PL concentration of 5000 μg/mL) in the conditions chosen according to the MIC and MBC data, *Staphylococcus aureus* adhered to each other, and the boundaries between the bacteria became blurred. However, the bacteria body still remained intact (Figure 3B,C). Figure 3D shows that the surface of untreated *Bacillus subtilis* was smooth, full, and complete, and the bacteria body was short rod-shaped. After ε-PL treatment, wrinkles and holes appeared on the surface of *Bacillus subtilis*, and the short rod-shaped structure was destroyed due to the deformation of the bacteria body (Figure 3E). Similarly, after treatment with the complexes, large areas of voids appeared on the surface of *Bacillus subtilis*, and the shape of the bacteria body was completely destroyed (Figure 3F). The results of SEM confirmed the antibacterial effect of whey protein-ε-PL complexes on *Staphylococcus aureus* and *Bacillus subtilis*. ε-PL has shown both bacteriostatic and bactericidal properties [41]. For the bacteriostatic properties, ε-PL could be combined with the bacterial membrane, resulting in bacterial membrane sub-damage, and bacteria could repair this sub-damage through their own repair mechanism to maintain normal growth and reproduction [3,42]. The results in this study found that the complexes (1:1 mass ratio of whey protein-to-ε-PL with a final ε-PL concentration of 5000 μg/mL) could destroy the cell membrane structure of *Bacillus subtilis*, resulting in the holes that appeared on the surface, but not for *Staphylococcus aureus*.

### 3.5. MD Simulation

MD simulation can analyze peptides at the atomic level, thus helping to further understand the antibacterial mechanism of substances. The mechanism of bacterial inhibition of whey protein-ε-PL complexes in monolayer and bilayer models was investigated using MD simulation [24].

#### 3.5.1. Schematic Diagram of Bacteriostatic Model of Bilayer Membrane

**Figure 4:**
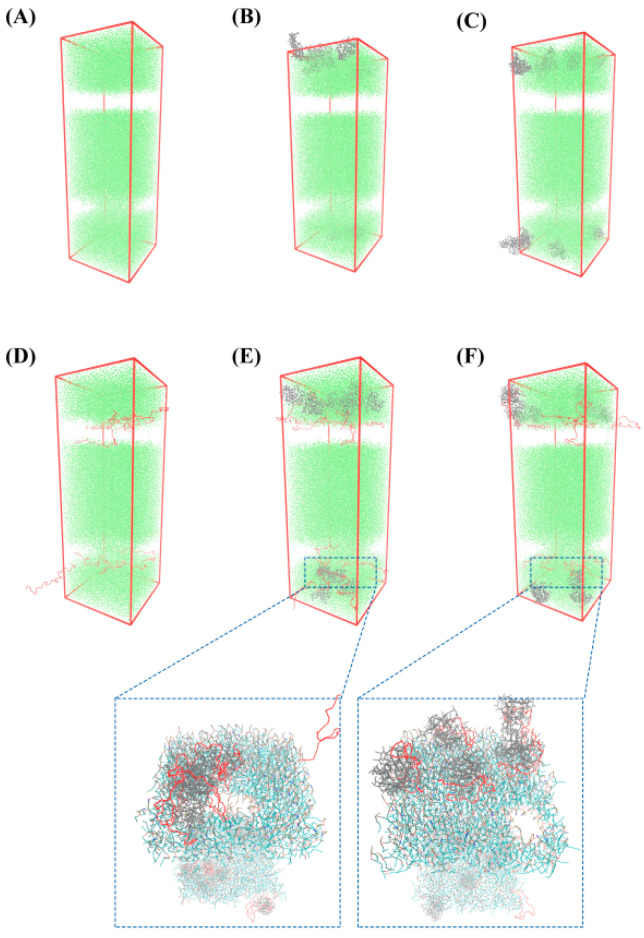
The interaction diagram of ε-PL, whey protein, and the whey protein-ε-PL complexes with the bilayer membrane model of Gram-positive bacteria. (**A**) POPE/POPG. (**B**) α-La-POPE/POPG. (**C**) β-Lg-POPE/POPG. (**D**) ε-PL-POPE/POPG. (**E**) ε-PL-α-La-POPE/POPG. (**F**) ε-PL-β-Lg-POPE/POPG.

As shown in Figure 4A, the bilayer membrane of Gram-positive bacteria could maintain an intact configuration and could be used as a reference group. In the systems α-La-POPE/POPG (Figure 4B) and β-Lg-POPE/POPG (Figure 4C), after the MD simulation, α-La and β-Lg did not adsorb to the surface of the bilayer membrane of Gram-positive bacteria. The results indicated that α-La and β-Lg could not interact with the bilayers of Gram-positive bacteria and affect the integrity of the bilayers. In the system ε-PL-POPE/POPG (Figure 4D), after the MD simulation, parts of the ε-PL molecules could be embedded in the bilayer membrane, resulting in the formation of pores, which would connect the internal and external environment of bacteria. Meanwhile, parts of the ε-PL molecules could penetrate the bilayer membrane and then enter the bacterial internal environment, which may lead to the disruption of bacterial growth. In the systems ε-PL-α-La-POPE/POPG (Figure 4E) and ε-PL-β-Lg-POPE/POPG (Figure 4F), similar results were observed. The ε-PL molecules of the complexes could be embedded in the bilayer membrane or pass through the bilayer membrane into the bacterial internal environment, which could destroy the structure of the bacterial membrane and result in bacterial death.

#### 3.5.2. Analysis of *Z*-Axis Potential Difference of Bilayer Membrane

**Figure 5:**
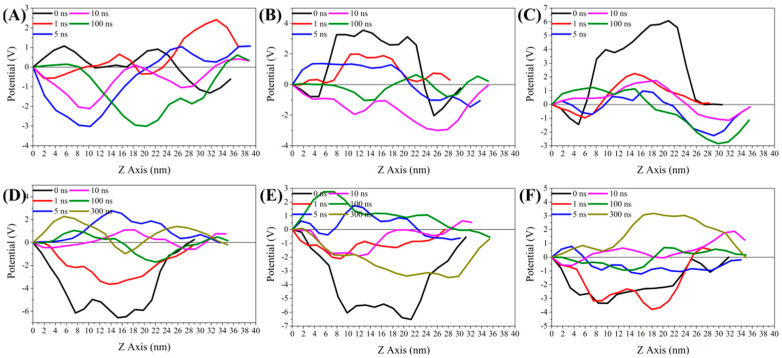
The variation curve of the *Z* axis potential difference in the interaction of the ε-PL, whey protein, and the whey protein-ε-PL complexes with the Gram-positive bacteria bilayer membrane model. (**A**) POPE/POPG. (**B**) α-La-POPE/POPG. (**C**) β-Lg-POPE/POPG. (**D**) ε-PL-POPE/POPG. (**E**) ε-PL-α-La-POPE/POPG. (**F**) ε-PL-β-Lg-POPE/POPG.

The MD simulation results of Figure 5 were controlled by the potential difference between the external environment and the internal environment of the bilayer membrane. As shown in Figure 5, at the beginning of the MD simulation (0 ns), there existed a potential difference between the external environment and bilayer membrane center (*Z* axis: 17–20 nm) during the system POPE/POPG, α-La-POPE/POPG, β-Lg-POPE/POPG, ε-PL-POPE/POPG, ε-PL-α-La-POPE/POPG, and ε-PL-β-Lg-POPE/POPG. In the MD simulation, the potential difference in all scenarios would result in the formation of hydrophilic pores in the bilayer membrane. Meanwhile, the ε-PL molecules could bind to the negatively charged phospholipid bilayer. Parts of the ε-PL molecules that were bound near the hydrophilic holes had the ability to gradually approach the holes by interaction, and then pass through the holes into the internal bacterial environment. However, the α-La molecules and β-Lg molecules had the same electric charge as the phospholipid membrane molecules, which would prevent the α-La and β-Lg molecules from getting close to the hydrophilic holes and being embedded in the bilayer membrane or entering the bacterial internal environment. When exploring the mechanism of action of whey protein-ε-PL complexes through MD simulation, it was likewise found that the compounds were able to form nano-sized annular holes on the DPPC membrane [43]. Some researchers concluded that antimicrobial peptides have the ability to interact with the amphiphilic membrane DMPC through the annular holes by means of MD simulation, for example, the linear amphipathic α-helical antimicrobial peptides [44]. These results were similar to the results of the present study.

#### 3.5.3. Density Distribution Curve of Components in Bilayer Membrane System

**Figure 6:**
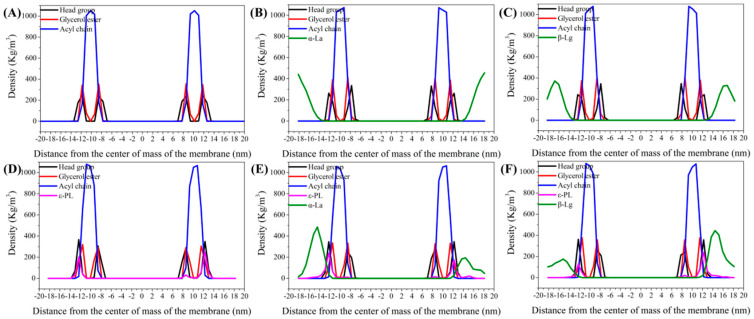
The *Z*-axis density distribution curve of the components in the interaction of the ε-PL, whey protein, and the whey protein-ε-PL complexes with the Gram-positive bacteria double membrane model. (**A**) POPE/POPG. (**B**) α-La-POPE/POPG. (**C**) β-Lg-POPE/POPG. (**D**) ε-PL-POPE/POPG. (**E**) ε-PL-α-La-POPE/POPG. (**F**) ε-PL-β-Lg-POPE/POPG.

In order to analyze the specific sites where the whey protein-ε-PL complexes interacted with the bilayer membrane, the density distribution curve of the components of all MD systems was calculated after MD simulation (Figure 6). The membrane molecule was divided into head group, glycerol ester, and acyl chain to observe the insertion sites of whey protein-ε-PL complexes, ε-PL, and whey protein in the membrane. In Figure 6, the X-axis 0 represents the center of mass of the bilayer membrane.

Figure 6B,C show that α-La and β-Lg were located in the external bilayer environment and there was no direct interaction between the whey protein and bilayer membrane. In Figure 6D–F, parts of the ε-PL density curves overlap with the density distributions of the external and internal membrane head group, glyceryl ester, and acyl chain. These results are consistent with the fact that the ε-PL molecules could be embedded in the bilayer membrane model or pass through the bilayer membrane into the inner bacterial environment (Figure 4).

### 3.6. Antibacterial Effect of Whey Protein-ε-PL Complexes in Fresh Meat

**Figure 7:**
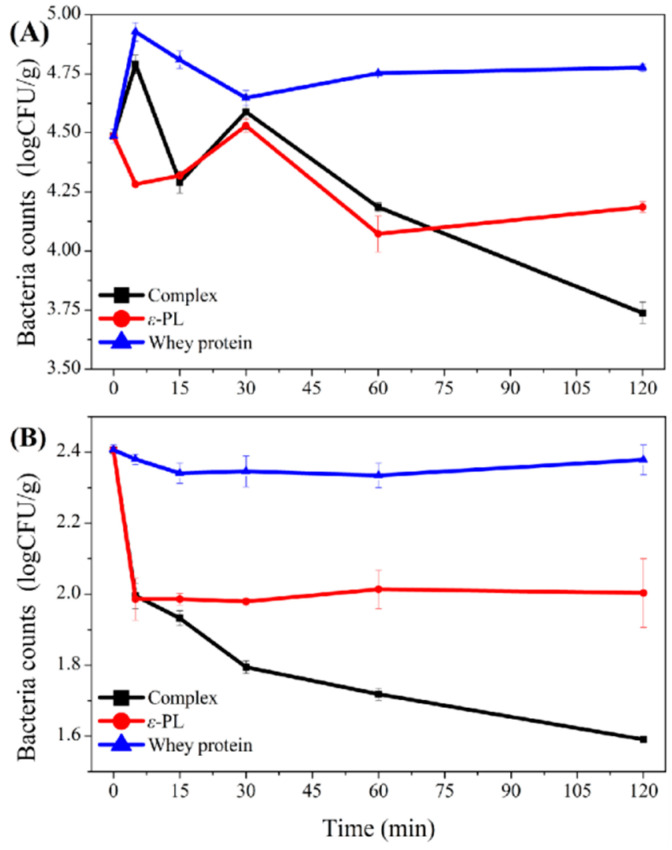
Bactericidal effect over time of whey protein-ε-PL complexes, ε-PL, and whey protein on (**A**) total mesophilic and (**B**) native *Staphylococcus aureus* counts in fresh meat.

The antimicrobial effect of the whey protein-ε-PL complexes on total mesophilic counts and native *Staphylococcus aureus* was investigated in fresh meat (Figure 7). According to a preliminary experiment, *Bacillus subtilis* was not detected in fresh meat. There was a significant decrease in the number of bacteria over time by the complexes treatment (1:1 mass ratio of whey protein-to-ε-PL with a final ε-PL concentration of 5000 μg/mL, 120 min). As shown in Figure 7A, the total mesophilic count was reduced to 3.74 log CFU/g in the sample treated with the complexes for 120 min while ε-PL reached 4.19 log CFU/g and the negative control was 4.78 log CFU/g. As shown in Figure 7B, the results indicated that the *Staphylococcus aureus* count was reduced to 1.59 log CFU/g in the sample treated with the complexes for 120 min while ε-PL reached 2.00 log CFU/g and the negative control was 2.38 log CFU/g. These results confirmed the bactericidal effect of the whey protein-ε-PL complexes in actual food systems (fresh meat) and that such an effect was larger than using ε-PL alone. It was hypothesized that ε-PL tended to bind to anionic proteins in fresh meat to produce precipitation, thus affecting its antibacterial effect. In contrast, the whey protein-ε-PL complexes better balanced its solubility and efficient antibacterial activity, prevented the binding of ε-PL to anionic proteins in fresh meat, and therefore had a better bactericidal effect. This agrees with our previous work on ε-PL and macromolecules (i.e., sodium tripolyphosphate and carboxymethyl chitosan) complexes, which were proven to have a similar antibacterial activity to ε-PL and exhibited an excellent bacteriostatic effect in food systems [45,46].

## 4. Conclusions

The interaction between ε-PL and whey protein did not diminish the efficient antibacterial properties of the complexes against *Staphylococcus aureus* and *Bacillus subtilis*. The complexes had both bacteriostatic and bactericidal effects. Among the treatments tested, the complexes against microorganisms showed the highest antibacterial activity at the mass ratio of whey protein-to-ε-PL of 1:1. The whey protein-ε-PL complexes could disrupt the structure of the cell membrane in *Bacillus subtilis*, creating holes on the bacterial surface, but not in *Staphylococcus aureus*. The antibacterial mechanism of the whey protein-ε-PL complexes was also revealed: the complexes and bacterial membrane were approached through electrostatic attraction. The complexes then affected the interaction between the membrane molecules through hydrogen bonding between the amino group of ε-PL and the oxygen atom of the membrane head group. Finally, parts of the ε-PL molecules could be embedded in the bilayer membrane, forming pores that could connect the internal and external environment of bacteria. Meanwhile, parts of the ε-PL molecules could penetrate the bilayer membrane and enter the internal environment of bacteria, which may lead to the disruption of bacterial growth and result in bacterial death. Additionally, the whey protein-ε-PL complexes could inactivate microorganisms in fresh meat with better antibacterial activity than ε-PL. This study confirmed the antibacterial activity of whey protein-ε-PL complexes against Gram-positive bacteria and provided a theoretical basis for its antibacterial mechanism.

## Figures and Tables

**Table 1:** Components and properties of whey protein.

Composition	Typical	Specification	Test Method
Protein (% dry basis)	93.0	91.0 min	Calculation
Protein (% as is)	89.0	87.0 min	AOAC
Lactose (%)	0.1	/	AOAC
Fat (%)	1.3	1.8 max	AOAC
Moisture (%)	4.7	6.0 max	AOAC
Ash (%)	2.7	3.5 max	AOAC
pH	/	6.2–7.0	10% Sol. At 20 °C

**Table 2:** The parameters of the bilayer membrane molecular dynamics (MD) simulation.

System ^a^	ε-PL (Number)	Protein (Number)	POPE/POPG	Temperature (°C)	Time (ns)
POPE/POPG	0	0	1024	37 °C	100
α-La-POPE/POPG	0	3	1024	37 °C	100
β-Lg-POPE/POPG ε-PL-POPE/POPG	0 10	10 0	1024 1024	37 °C 37 °C	100 300
ε-PL-α-La-POPE/POPG	12	3	1024	37 °C	300
ε-PL-β-Lg-POPE/POPG	10	10	1024	37 °C	300

^a^ POPE represents 1-Palmitoyl-2-Oleoyl-sn-Glycero-3-Phosphoethanolamine, POPG represents 1-Palmitoyl-2-Oleoyl-sn-Glycero-3-Phosphoglycerol, ε-PL represents ε-polylysine, α-La represents α-lactalbumin, β-Lg represents β-lactoglobulin. The molecular ratio of POPE and POPG in the double-layer membrane model of Gram-positive bacteria is 1:3 and thee POPE/POPG value is 1024 according to the model for biomolecular simulations [31].

**Table 3:** The minimum inhibitory concentration (MIC) of whey protein, ε-PL, and whey protein-ε-PL complexes against *Staphylococcus aureus* and *Bacillus subtilis*.

Samples	Mass Ratios (Whey Protein-to-ε-PL)	MIC (μg/mL)	
		*Staphylococcus aureus*	*Bacillus subtilis*
whey protein	/	-	-
ε-PL	/	19.53	4.88
complexes	50:1	25.00	6.25
complexes	50:2	25.00	6.25
complexes	50:10	31.26	7.81
complexes	50:20	31.26	3.90–7.81
complexes	50:30	23.44	5.86
complexes	50:40	31.26	3.90
complexes	50:50	19.53	4.88

**Table 4:** The minimum bactericidal concentration (MBC) of whey protein, ε-PL, and whey protein-ε-PL complexes against *Staphylococcus aureus* and *Bacillus subtilis*.

Samples	Mass Ratios (Whey Protein-to-ε-PL)	MBC (μg/mL)	
		*Staphylococcus aureus*	*Bacillus subtilis*
whey protein	/	-	-
ε-PL	/	39.06	9.77
complexes	50:1	50.00–100.00	12.50–25.00
complexes	50:2	50.00–100.00	12.50–25.00
complexes	50:10	62.50	15.63
complexes	50:20	62.50	15.63
complexes	50:30	93.75	11.72–23.43
complexes	50:40	62.50	15.63
complexes	50:50	39.06–78.13	9.77

## Data Availability

The data used to support the findings of this study can be made available by the corresponding author upon request.
